# Association of decreased mitochondrial DNA content with the progression of colorectal cancer

**DOI:** 10.1186/1471-2407-13-110

**Published:** 2013-03-12

**Authors:** HaiHong Cui, Ping Huang, ZhiJing Wang, YunXin Zhang, ZhenHua Zhang, Wei Xu, XiaoPeng Wang, Ying Han, XiaoMing Guo

**Affiliations:** 1 Department of Gastroenterology, 305 Hospital of PLA, Beijing, China; 2 Department of Gastroenterology, Beijing Military General Hospital of PLA, Beijing, China; 3 Department of Pathology, Institute of radiation medicine, Beijing, China

**Keywords:** Colorectal cancer, Mitochondrial DNA, Copy number, Quantitative PCR, Prognosis

## Abstract

**Background:**

Experimental data suggest that mitochondria is involved in tumorigenesis. However, little is known about the qualitative and quantitative changes of mtDNA in colorectal cancer tissues. We therefore conducted possible correlations of the mitochondrial DNA (mtDNA) copy number in colorectal cancer (CRC) with clinical and pathological findings and CRC prognosis.

**Methods:**

mtDNA copy numbers in CRC cancer tissue and adjacent non-cancerous tissue samples were measured using quantitative real-time polymerase chain reaction analyses from 60 patients admitted to our hospital. We examined the correlation of mtDNA copy numbers and clinicopathologic parameters of CRC patients. The correlation between mtDNA copy number and three-year survival was analyzed.

**Results:**

The mtDNA copy number was lower in CRC tissue compared with the corresponding non-cancerous colorectal tissue (mean: 108.60 ± 20.11 *vs*. 153.68 ± 25.72) and was significantly correlated with lymph-node metastasis. Patients with a lower mtDNA copy number tended to have lower 3-year survival than patients with a higher mtDNA copy number assessed by Kaplan–Meier curves, but the correlation was not significant (overall survival, 63.0 *vs* 83%).

**Conclusions:**

These results suggest that a reduced copy number of mtDNA is correlated with malignant potential in CRC.

## Background

Mitochondria contain their own genetic systems. Mitochondrial DNA (mtDNA) is a 16.569-kb circular double-stranded molecule used for replication, transcription and translation [1]. mtDNA is present at thousands of copies per cell and varies in number according to cell type. mtDNA programs a small (12S) and large (16S) ribosomal RNA gene, 22 transfer RNAs and 13 protein-coding genes. Mitochondria are responsible for the supply of most of the energy needed by human cells, and have a key role in the initiation and execution of apoptosis. Moreover, mitochondria are the major intracellular producers of reactive oxygen species (ROS) and are subject to direct attack by ROS in the organelles of mammalian cells. Characteristics such as a lack of introns, inability to bind to histones, and inefficient mtDNA proofreading and DNA repair systems render mtDNA more susceptible to oxidative damage than nuclear DNA (nDNA) [2]. Impairment of mitochondrial respiratory function not only reduces the supply of energy (which may prevent energy-dependent apoptosis) but also enhances ROS production, which may induce mutation and oxidative damage to mtDNA. It has been reported that accumulation of mtDNA mutations as well as alteration in the execution of apoptosis contribute to the onset and progression of various myopathies. Recently, it was reported that there are common mechanisms by which the bioenergetic function of mitochondria is altered in cancer cells. However, little is known about the qualitative and quantitative changes of mtDNA in the alteration of mitochondrial oxidative phosphorylation in colorectal cancer tissues.

Colorectal carcinoma (CRC) is the third most prevalent cancer and the second highest cause of cancer death in the world [3]. The life expectancy of individuals with CRC is mainly dependent on the clinical stage at which CRC is detected. Chemotherapeutic regimens can only marginally improve the prognosis of advanced cases [4]. To achieve better outcomes for patients, it would be desirable to identify and target cellular molecules involved in the carcinogenesis of CRC. However, the effect of CRC on mtDNA copy number is unclear. In the present study, we investigated the correlation of mtDNA copy number with the clinicopathologic features and prognosis of CRC.

## Methods

Informed consent was obtained from each patient according to the protocols approved by the ethics committees of 305 Hospital of PLA (Beijing, China). Specimens of CRC (as well as the corresponding normal margin) from 60 CRC patients diagnosed and treated in our hospital from January to October 2008 were retrieved from the hospital’s pathology department by two pathologists. All the patients were not receiving preoperative radiotherapy and/or chemotherapy, age 22 to 81 years, with an average of 56.7 years old. 30 males and 30 females. I stage six cases, II stage 32 cases, III stage 14 cases, IV stage eight cases (NCCN Classification Standard); well differentiated 17 cases, differentiated 31 cases, poorly differentiated 12 cases.

### DNA extraction

To obtain pure tumor cells from cancerous tissues, laser capture microdissection (LCM) was used. Each paraffin block was cut at a thickness of 10 μm into 5 slices. Genomic DNA was extracted using a QIAamp DNA Mini Kit (Qiagen, Berlin, Germany).

### Real-time quantitative polymerase chain reaction (PCR) analyses

A precise assay based on fluorogenic real-time quantitative PCR was developed to compare the relative abundance of mtDNA with nuclear DNA. The sequences of primers used for amplification of ND1 gene in mtDNA were: forward, 5^′^-TAATGCTTACCGAACGAA-3^′^, reverse, 5^′^-TTATGGCGTCAGCGAAGG -3^′^, 104 bp. The sequences of primers used for amplification of the β-actin gene in nuclear DNA were: forward, 5^′^-GCAAAGTTCCCAAGCACA-3^′^, reverse, 5^′^-AAGCAAGCAGCG GAGCAG-3^′^, 105 bp. The PCR conditions were: hot start at 95°C for 5 min, followed by 40 cycles of 95°C for 30 s, 57°C for 45 s, and 72°C for 45 s. The PCR was carried out for 40 cycles in a 50 μL reaction mixture containing 200 ng DNA, 200 μM of dNTP, 40 pmol of each primer, 1.0 U of Taq DNA polymerase, 50 mM KCl, 1.5 mM MgCl2, 10 mM Tris–HCl (pH 9.0), 0.1% Triton X-100, and 0.1% (w/v) gelatin. The PCR products were separated by electrophoresis on a 3% agarose gel at 100 V for 40 min and detected under UV transillumination after ethidium bromide staining. The products were cleared by QIAquick PCR Purification Kit(QIAGEN Germany), which were combined with pMD 18-T Vector(program USA), a standard curve was presented by recombinant plasmids serially diluted 10^4^ to 10^8^ times. The fluorescence intensity was measured at the end of each extension phase at 72°C. The absolute copy number of the target and internal standard DNAs was analyzed with real-time quantitative polymerase chain reaction(RT-PCR) using the SYBR Green method (MJ Research Instrument, USA), The real-time PCR conditions were: 95°C denaturation for 5 min, 40 cycles of 95°C for 10 s, 60°C for 10 s, 68°C for 20 s. The PCR was carried out for 40 cycles in a 10 μl reaction mixture containing Sybergreen-I 0.5 μl, 10 × buffer 1 μl, dNTP Mixture 0.25 μl, primer, 2 μl, standard or sample 1 μl of ddH2O 5.25 μl. The DNA content of the ND1 gene was normalized with that of the β-actin gene in nuclear DNA to calculate the copy number of mtDNA in each sample.

### Determination of mtDNA copy number

**Figure 1:**
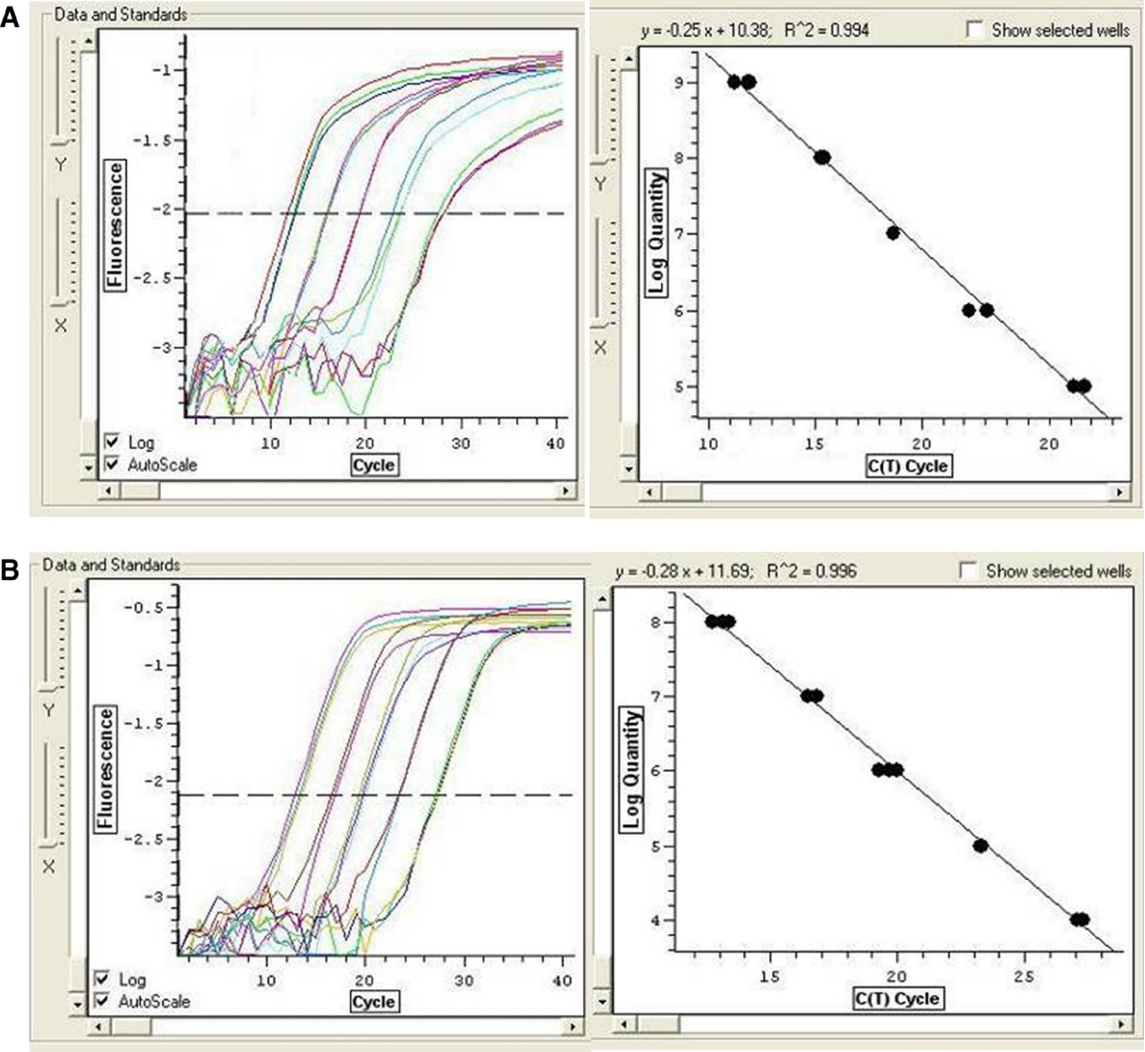
**A ND1 quantitative PCR amplification of the power curve in the standard and sample. B** β-actin quantitative PCR amplification of the power curve in the standard and sample.

The nuclear gene β-actin was chosen as the internal reference for quantifying mtDNA copy number and as a marker of diploid genome content. ND1 represents the mtDNA copy number. Standard curves relating mtDNA and nDNA copies to replication threshold cycles (Ct) were established using total cellular DNA of CRC tumorous cells (Figure 1). For each clinical sample, the Ct values for mtDNA and nDNA were determined and their mtDNA and nDNA amounts relative to CRC tumorous cells plotted and corrected from the standard curve. Using the β-actin gene as the internal standard (whose mtDNA copy number (mtDNA amount/nDNA amount) was defined as 1), the relative mtDNA copy numbers of the 60 clinical samples were calculated [5]. The same test was duplicated and mean copy numbers and standard errors calculated.

### Statistical analyses

The chi-square test and non-parametric test were used to examine the association between mtDNA copy number and the age and sex of the patient, pathological type of CRC sample, clinical stage, and metastases to lymph nodes. Kaplan–Meier and log-rank methods were used to estimate survival. Statistical significance was set at p < 0.05.

## Results

### Generation of standard curves

Standard curves of mtDNA ND1 and the β-actin gene quantification assay are shown in Figure 1A and B. The regression line was based on five values ranging from 10^4^ DNA copies to 10^8^ DNA copies, which integrated all separate PCR runs and showed good linearity. The correlation coefficient (r2) was 0.994 and 0.996 for the standard curve of mtDNA ND1 and β-actin respectively, which suggested an acceptable degree of precision for our quantitative method.

### mtDNA copy number of CRC

To evaluate if changes in the quantity of mtDNA occurred within the tumor tissues of CRC patients, we analyzed the mtDNA copy number of tumor tissues and corresponding non-cancerous colorectal tissues by real-time quantitative PCR. Forty-two cases of CRCs (70%) showed reduction of mtDNA copy number compared with non-cancerous colorectal tissues. The mean mtDNA copy number in each cell equaled 2ND1/β-actin and was significantly lower than that in non-cancerous colorectal tissues. The mean copy number of mtDNA was 108.60 ± 20.11 in tumors and 153.68 ± 25.72 in matched non-cancerous tissue, respectively.

To further explore the correlation between mtDNA copy number and the clinicopathological variables of CRC, we calculated the ratio of the copy number in tumors to that in paired normal tissues. This was designated as the T/N ratio (mtDNA copy number/β-actin in T divided by mtDNA copy number/β-actin in N). Based on the different T/N ratios, patients were categorized into two subgroups according to the median value of the T/N ratios (0.72). The association between mtDNA quantitative change and the clinicopathological parameters are summarized in Table 1. Patients with lymph-node metastasis were more likely to have a lower T/N ratio compared with patients without lymph-node metastasis, (p < 0.05). However, there was no significant correlation between the lower T/N ratio and other characteristics, including sex, age, and tumor, node, metastasis (TNM) stage.

**Table 1:** Relationship between change in mtDNA copy number and clinicopathological parameters

**Clinico-pathological parameter**	**Group**	**Number (n)**	**Low mtDNA copy number (n)**	**High mtDNA copy number (n)**	***Χ***^**2 **^**and Z**	**P**
Sex	Male	30	17	12	1.669	0.196
	Female	30	13	18		
Age (years)	<57	30	16	13	0.601	0.438
	≥57	30	14	17		
Pathological stage	Well differentiated	17	5	7	−1.321	0.186
	Moderately differentiated	31	14	17		
	Poorly differentiated	12	11	6		
Lymph node metastasis	Positive	17	12	5	4.022	0.045*
	Negative	43	18	25		
TNM stage	I	6	2	4	−1.604	0.109
	II	32	14	18		
	III	14	9	5		
	IV	8	5	3		

* It indicated that the low mtDNA copy number correlated significantly with lymph node metastasis group (P=0.045, Table 1).

### Association of mtDNA copy number and patient survival

**Figure 2:**
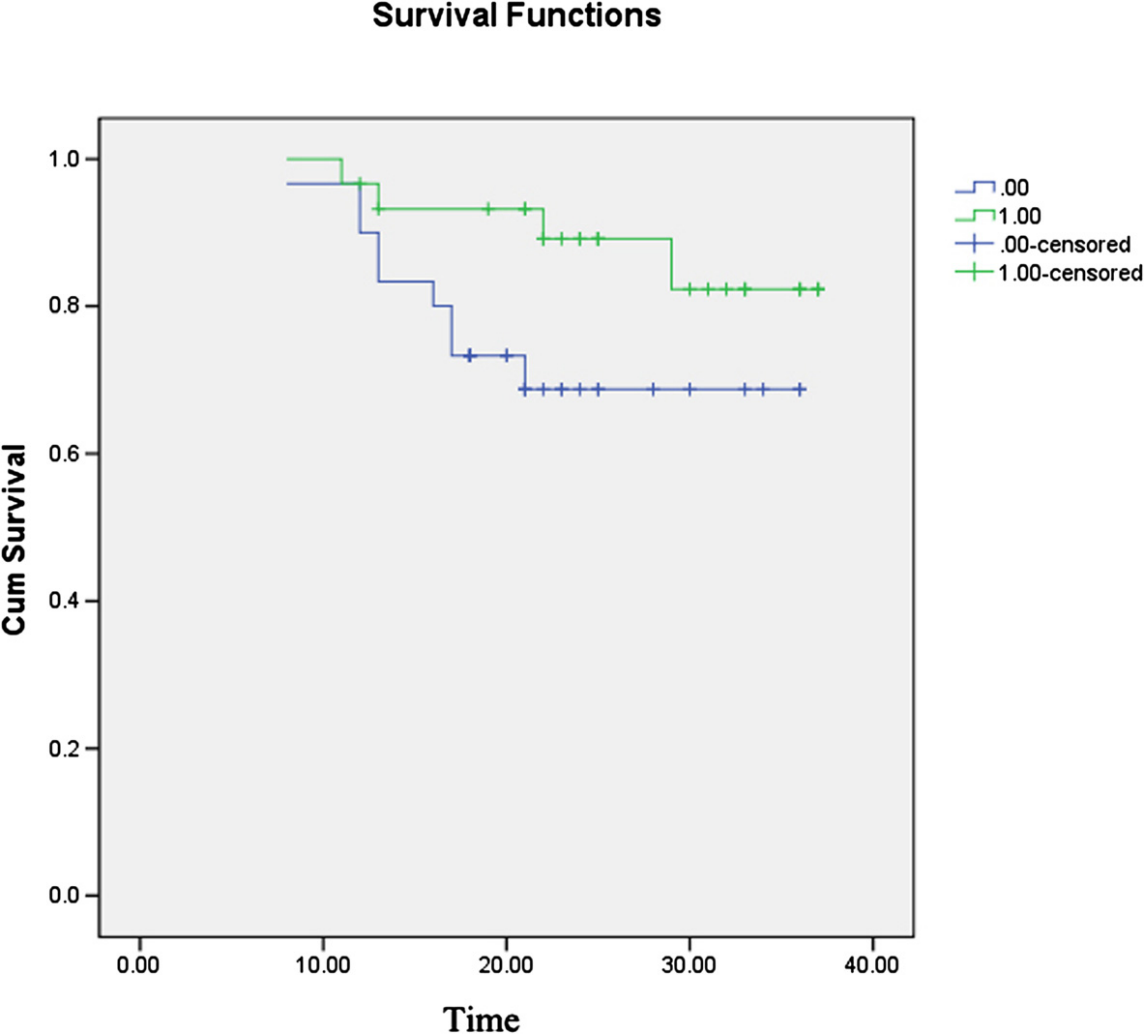
**Overall survival.** Patients who had a lower mtDNA copy number had poorer three-year survival than patients with a higher mtDNA copy number when assessed by Kaplan–Meier curves, but the difference was not significant (overall survival, 70.0 *vs* 86.7%, P = 0.082). 0 = low mtDNA copy number group; 1 = high mtDNA copy number group.

We then assessed the overall prevalence of survival to assess the prognostic significance of mtDNA copy number. The three-year overall survival of 60 patients with CRC was 78.3%. Kaplan–Meier curves revealed that patients who had a lower mtDNA copy number (T/N ratio <0.72) tended to have poorer three-year survival than patients who had a higher mtDNA copy number (T/N ratio >0.72) but the difference was not significant. (overall survival, 70.0 *vs* 86.7%; p = 0.082, Figure 2).

## Discussion

Mitochondria are cellular organelles bounded by two distinct membranes. They contain one-tenth of the cellular proteins. Their DNA replicates autonomously and is transmitted through the maternal germline independently of nuclear and chromosomal DNA [6]. Each cell in the human body contains up to several hundred mitochondria. Each mitochondrion contains ≤10 copies of mtDNA. In addition to producing energy, mitochondria support and participate in several essential cellular functions, including: intermediary metabolism; ion homeostasis; synthesis of lipids, amino acids and nucleotides; active transport; and apoptosis [7]. The aging process in humans has been shown to be associated with reduced levels of mtDNA transcripts and increased mtDNA content in the brain and lung [8,9]. There are undoubtedly tissue-specific effects of aging because the skeletal muscles and livers of rats showed an age-related decline in mtDNA copy number. Different from nuclear DNA, replication of mtDNA is error-prone and mammalian mtDNA contains no introns, has no protective histones, and is exposed to deleterious ROS generated by oxidative phosphorylation [10]. These factors contribute to the accumulation of mutations in mtDNA at an approximately tenfold greater rate than in nuclear DNA [11]. The mtDNA of tumor cells may not only suffer from the change of structure but also the change of number. Some studies have shown that copy number is increased in tumors. Wang et al. found that the average mtDNA copy number in pathological low-grade tumours was over two-fold higher than that in high-grade endometrial carcinomas, and the Change in mtDNA content was not related with patients’ age or tumour stages [12]. The copy number of 1p36.33 and mtDNA peripheral blood mononuclear cells infected by the Epstein–Barr virus in patients with lymphocytic leukemia was tested in quantitative PCR by Jeon et al. They suggested that increased mitochondrial biogenesis is indicative of the progression of EBV-mediated B-cell transformation [13]. However, some studies showed that the mtDNA copy number was decreased in tumors. Lee et al. found: a marked decrease in cellular mtDNA and ATP content, concomitant with a lack of mRNAs encoded by mtDNA. The mtDNA- depleted cells showed a decreased sensitivity and accumulation of anti-cancer drugs, suggesting that mtDNA depletion could develop multidrug resistance (MDR) phenotype in HCT-8 cells. The expression level of MDR1 mRNA and its translated product P-glycoprotein was increased in the mtDNA- depleted cells. The decline in mtDNA content may cause multidrug resistance, which may be raised by increasing the stability of MDR1 mRNA expression [14]. Lee et al. examined three colon cancer cells and found that the decline in mtDNA copy number may be independent of tumor-cell homogeneity or heterogeneity of point mutations or large fragment deletion. In human tumors, the instability of the mitochondrial genome and the decline of mtDNA copy number may be independent factors [15]. Decline in mtDNA copy number may be related to the reduction in the number of oxidative phosphorylation proteins. In various tumors, reduction of mtDNA copy number may be correlated with clinicopathological parameters and tumor invasiveness, For example, compared with normal liver tissues, the mtDNA copy number in hepatocellular carcinoma(HCC) was significantly reduced, and the results showed that it was related to tumor size and cirrhosis. It is evident that a low mtDNA copy number in HCC patients might help to identify a poor prognosis [16]. Scientists could establish cell lines containing varying levels of mtDNA by treatment with low concentrations of ethidium bromide. Ethidium bromide can insert into mtDNA molecules and inhibit the activity of mtDNA polymerase gamma during mtDNA replication. Amuthan and colleagues demonstrated that an invasive phenotype is produced within the original cell line of the non-invasive mouse skeletal muscle C1C12 cell line and human lung cancer cell line A549 if mtDNA is absent, and that the overexpression of the tumor-specific markers cathepsin L and β-transforming growth factor can be detected. These studies suggest that the loss of mtDNA can promote the development and metastasis of tumors [17]. The loss of mtDNA may affect the development of cancer and cancer metastasis by preventing apoptosis and promoting the generation of cancer-related proteins. Wu et al. suggest that somatic mtDNA mutations and mtDNA depletion occur in gastric cancer and that mtDNA depletion is involved in carcinogenesis and/or cancer progression of gastric carcinoma [18]. Some evidences suggest that a loss in the integrity of the mitochondrial genome (sequence and/or copy number) can progress a cancer to an advanced phenotype by way of ROS dependent and independent events [19].

Tumor growth requires an appreciable amount of energy, which is mainly from mitochondrial aerobic oxidation and glycolysis, which is required for excessive cell proliferation. Previously, it was believed that long-term hypoxia as well as glycolysis in the tumor played a leading part in the rapid growth of tumors. Recently, it was found that these two types of energy supply in tumor growth have equally important roles. Mitochondria are the only gene-containing structures outside the nucleus in eukaryotic cells. The rate of somatic mutation of mtDNA has been presumed to be 10–100-times greater than that of nuclear DNA. Reported sequence changes include point mutations, multiple deletions and microsatellite instability in coding and non-coding regions. However, little is known about rapid decreases in mtDNA, microsatellite instability and alteration of the copy number in human CRC.

We found that the mean copy number in CRC was significantly lower than the copy number in adjacent tissues. The copy numbers obtained from the present study were significantly lower than the copy number of myocardial and peripheral blood mononuclear cells reported from other studies [5,20]. We also found that the decrease in mtDNA copy number was significantly correlated with lymph-node metastasis but not with sex, age, pathological type, or TNM stage. We also found that the three-year tumor-free survival was lower among CRC patients with lower mtDNA copy numbers. This may be because the low copy numbers in tumors result in a stronger tolerance to hypoxia. Therefore, tumors can reduce the dependence of mitochondrial oxidative phosphorylation. Hence, the main source of energy for tumor progression is rendered to anaerobic metabolism in the glycolytic pathway. This is a favorable measure for tumor invasion, and which is a necessary condition for survival of the tumor under hypoxic conditions [21]. Tumors with relatively high copy numbers of mtDNA have poor tolerance to hypoxia grow slowly, they are less invasive, and more sensitive to chemotherapy. Thus, patients with such tumors have a better prognosis [22], Tumors that metastasize to lymph nodes are more likely to recur even after surgical removal. We found that the copy number of mtDNA was negatively correlated with lymph-node metastasis. Patients with a relatively high copy number of mtDNA had a higher three-year tumor-free survival than patients with a lower copy number of mtDNA. but the difference was not significant. The small sample size might limit the significance of our study. A future should focus on a functional analysis of the reduction of mtDNA in CRC.

## Conclusions

In conclusion, these results suggest that a reduced copy number of mtDNA is correlated with malignant potential in Colorectal Cancer and the copy number of mtDNA was negatively correlated with lymph-node metastasis.

## Competing interests

The authors declare that they have no competing interest.

## Authors’ contributions

HHC, PH, YH, and YXZ conceived the study idea. All authors reviewed the literature and/or designed the study. HHC, PH, ZHZ, WX, XPW carried out the molecular genetic studies, ZHZ, WX, PXW analyzed the data. All authors interpreted the findings. HHC and PH organized the writing and wrote the initial draft. All authors edited the manuscript and approved the final version.

## Pre-publication history

The pre-publication history for this paper can be accessed here:

http://www.biomedcentral.com/1471-2407/13/110/prepub
